# Resilience of Alternative States in Spatially Extended Ecosystems

**DOI:** 10.1371/journal.pone.0116859

**Published:** 2015-02-25

**Authors:** Ingrid A. van de Leemput, Egbert H. van Nes, Marten Scheffer

**Affiliations:** Department of Environmental Sciences, Wageningen University, Wageningen, The Netherlands; University of Leeds, UNITED KINGDOM

## Abstract

Alternative stable states in ecology have been well studied in isolated, well-mixed systems. However, in reality, most ecosystems exist on spatially extended landscapes. Applying existing theory from dynamic systems, we explore how such a spatial setting should be expected to affect ecological resilience. We focus on the effect of local disturbances, defining resilience as the size of the area of a strong local disturbance needed to trigger a shift. We show that in contrast to well-mixed systems, resilience in a homogeneous spatial setting does not decrease gradually as a bifurcation point is approached. Instead, as an environmental driver changes, the present dominant state remains virtually ‘indestructible’, until at a critical point (the Maxwell point) its resilience drops sharply in the sense that even a very local disturbance can cause a domino effect leading eventually to a landscape-wide shift to the alternative state. Close to this Maxwell point the travelling wave moves very slow. Under these conditions both states have a comparable resilience, allowing long transient co-occurrence of alternative states side-by-side, and also permanent co-existence if there are mild spatial barriers. Overall however, hysteresis may mostly disappear in a spatial context as one of both alternative states will always tend to be dominant. Our results imply that local restoration efforts on a homogeneous landscape will typically either fail or trigger a landscape-wide transition. For extensive biomes with alternative stable states, such as tundra, steppe and forest, our results imply that, as climatic change reduces the stability, the effect might be difficult to detect until a point where local disturbances inevitably induce a spatial cascade to the alternative state.

## Introduction

The magnitude of a perturbation that a system can withstand without being tipped into an alternative stable state has been termed ‘ecological resilience’ by Holling [1]. Although the idea of alternative attractors and Hollings concept of resilience have become highly influential, most empirical work in ecology comes from relatively small, isolated systems, such as small lakes and ponds [2], from controlled isolated experiments [3,4], or from small enclosures in large-scale systems [5,6]. Here we address the fundamental problem of scaling up insights from such studies to spatially extended ecosystems. We consider a system spatially extended if the landscape is large relative to the scale of the relevant biological interactions, such that the system should not be considered well mixed.

Fundamental aspects of stability of spatially extended systems with local alternative states have been addressed so far mainly in theoretical literature from a rather abstract mathematical point of view [7]. Yet, the issue is highly relevant from a practical perspective in ecology. One may, for instance ask under which conditions local restoration efforts could flip a system to an alternative state that would remain stable despite an open connection to the rest of the landscape. Similarly, one may ask under which conditions climatic change would result in a patchwork of local shifts between alternative states such as forest and savanna [8], or invoke large scale synchronous shifts in tropical [9] or boreal biomes [10]. In essence, such problems boil down to the question under which conditions two alternative states may persist side by side in open connection.

**Fig 1:**
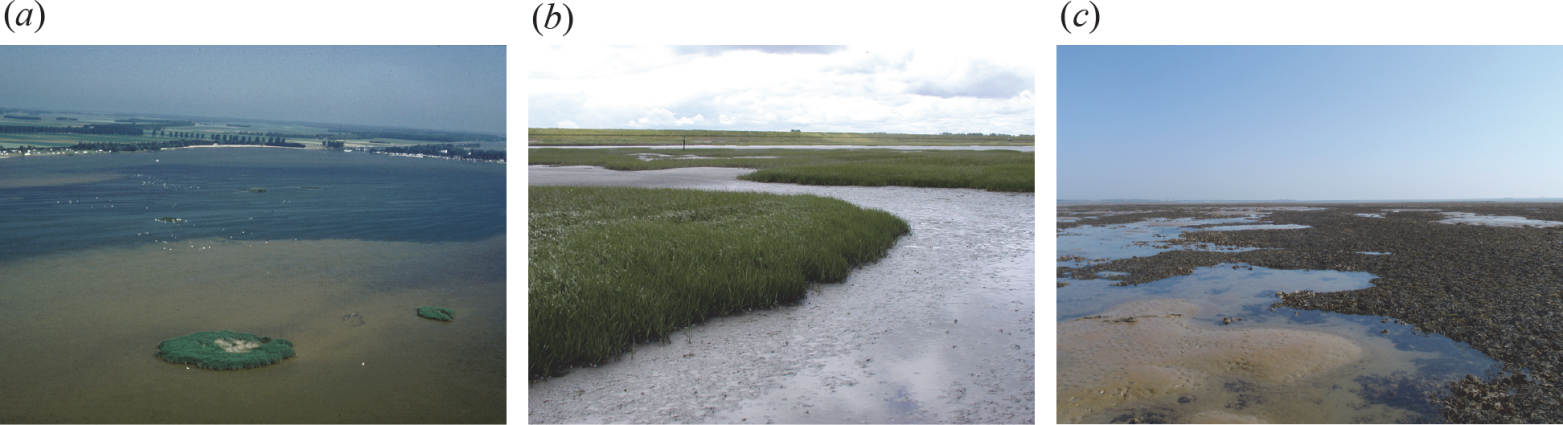
Example systems with alternative stable states in space. (*a*) Shallow lake: clear water with Chara vegetation vs. turbid water (photo by Ruurd Noordhuis). (*b*) Salt marsh: vegetation vs. bare marshland (photo by Johan van de Koppel). (*c*) Musselbed: mussels vs. bare soil (photo by Andre Meijboom).

This question has been specifically addressed in the context of invasion dynamics of species with a strong local Allee effect [11–13]. These modeling studies show that alternative stable states may co-exist side-by-side provided that the landscape consists of discrete units. Discrete units can clearly be distinguished in certain ecosystems, such as coral reefs connected through larval exchange [14,15] or shallow ponds connected through overflows [16,17]. However, some systems have contrasting states co-existing in apparently continuous and homogeneous landscapes (e.g. Fig. 1). For example, within shallow lakes we can find sharp boundaries between clear water with submerged plants, and turbid water with no vegetation [18] (Fig. 1*A*). In marshlands, patches of vegetation can be found adjacent to non-vegetated mudflat [19] (Fig. 1*B*). Distinct boundaries are also found between mussel beds and bare soil in intertidal zones [20,21] (Fig. 1*C*). Other examples include boundaries between forest, savanna and grasslands [22,23], or between kelp beds and bare ocean floor covered by sea urchins [24,25].

Could such apparent co-existence of alternative states be truly stable, or would it rather be a transient situation towards dominance by either of the states [19]? Clearly experimentation on relevant landscape scales is difficult, but there are even few spatially explicit theoretical studies on alternative stable states [7,26–30].

Here we use simple models in an effort to help narrowing the gap between our understanding of local alternative states and patterns in spatially extended systems. We systematically analyze the behavior of a spatially explicit model with local alternative stables states. We consider spatial exchange of the key species, and investigate the behavior of the system on a large landscape. First, we show how theoretical insights from physics literature as recently reviewed by Bel et al. [7] can help to understand resilience of spatially extended systems, defined as the capacity to recover upon a disturbance [1], and hysteresis, defined as the tendency of a system with alternative stable states to stay in the same state despite changes in external conditions [2]. Second, we investigate under which conditions alternative stable states may co-exist in space. We show how dispersal barriers and heterogeneity of other environmental conditions can lead to stable co-existence.

## Methods

### The model

Our model consists of a reaction-diffusion equation including a growth term (*f_N_ (N)*), and a spatially explicit diffusion term (*f_D_ (N,x)*) (Equation 1):

$$\frac{\partial N(x)}{\partial t}=f_{N}(N)+f_{D}(N,x)$$ (1)

For the growth term, we here use one of the simplest models with alternative stable states, the classical resource harvesting model. In this model, a resource species, *N*, is growing logistically, and is being harvested following a sigmoidal functional response (Equation 2).

$$f_{N}(N)=rN\left(1-\frac{N}{K}\right)-\frac{cN^{2}}{N^{2}+H^{2}}$$ (2)

The logistic growth is described by *r* as the maximal growth rate, and *K* as the maximum local biomass species *N* can reach. Mortality as a result of harvesting is described by *c* as the maximal mortality rate if *N* is high, and *H* as the half saturation level of the functional response. The modeled species can have two alternative stable states for a range of parameter settings: a low biomass state, for which high mortality rates prevent further growth, and a high biomass state, for which growth is limited by available food or space. In the non-spatial model, the size of the basin of attraction (i.e. ecological resilience) of each state varies with parameters such as the maximal mortality rate *c*, which we here assume to be the landscape-wide driver. Default parameters used for the growth-term are: *r* = 1 d^−1^, *K* = 10 g m^−1^, *c* = 2.4 g m^−1^ d^−1^, *H* = 1 g m^−1^. This model has been introduced as an overexploitation model [31,32]. More in general, it describes the dynamics of a population that has high per capita mortality rates at low biomass, and low per capita mortality rates at high biomass. Potential mechanisms for this are saturation of the predator, or decreased palatability or capture rate at high biomass. From a dynamical systems perspective the essence is the existence of a positive feedback mechanism that can cause a self-amplification of the effect of a disturbance around a critical threshold. For instance, if the biomass of the population in our model is depressed beyond a critical point, an increase in per capita mortality can lead to a further decrease and prevent the population to re-establish. We explored three other models in the supporting information (S1 Table) to show that our results are not specific to the model we use, as long as there is a local positive feedback that is strong enough to cause alternative stable states locally.

Space is represented by a one-dimensional continuum without discrete spatial units. Diffusion is the simplest form of modeling spatial exchange of the modeled species. As examples for spatial exchange one may think of clonal growth if the population represents vegetation, random movement if it represents a relatively sessile animal, or mixing if it represents water or dissolved nutrients (Equation 3):

$$f_{D}(N,x)=D\frac{\partial^{2}N}{\partial x^{2}}$$ (3)

with *D* as the diffusion rate, and *x* representing distance. This is the most straightforward way to model the scale at which feedbacks act. If *D* is high relative to the size of the landscape, local biomass differences are quickly smoothened out, such that feedbacks resulting from biomass differences practically affect the entire landscape. However, if *D* is low relative to the size of the landscape, local biomass differences remain for a relatively long period, such that the feedbacks act locally (see S1 Text for the non-dimensional form of the model, in which the spatial scale is scaled to the diffusion rate).

Additionally, we simulated dynamics on a heterogeneous landscape. We modeled heterogeneity in conditions, simply by assuming a landscape gradient of the growth rate *r*. We also simulated a landscape in which the diffusion rate (*D*) is varied randomly but smoothly in space. In order to simulate such smooth heterogeneous landscape, we first generated spatially auto-correlated stochastic diffusion rates on a one-dimensional grid of 30 cells (Equation 4).

$$D_{i+1}=0.3\cdot D_{i}+0.7\cdot D_{0}+Rnd_{i}$$ (4)

With *D*_*i*_ as the diffusion rate at grid cell *i*, *D*_*0*_ as the diffusion rate at *x* = 0, and *Rnd*_*i*_ as a random number from a normal distribution, with mean 0 and standard deviation *s*. Then, from these discrete values we made a landscape with smoothly varying diffusion rates over space using a Gaussian kernel smoothing function, by interpolating between these 30 cells [33] (Equation 5).

$$f_{D}(N,x)=\frac{\partial }{\partial x}\left(\left(\frac{1}{n}\sum_{i=1}^{30}G_{h}\left(x-x_{i}\right)\right)\frac{\partial N}{\partial x}\right)$$ (5)

With *G*_*h*_ as a Gaussian kernel, and *h* as the bandwidth of the kernel-smoothing window. Default parameters used for the diffusion equations are: *D* = 1 m^2^ d^−1^, *D*_*0*_ = 1 m^2^ d^−1^, *s* = 1.6 m^2^ d^−1^, *h* = 0.05 m.

### Computational approach

In order to test for the possibility of stable co-occurrence of alternative stable states in space, we initialized the studied landscape such that the left half of the landscape was in the high biomass state, and the right half in the low biomass state. To explore the effect of strong but local disturbances, we initialized the system such that the entire landscape was in one state. Then, we simulated a local disturbance by shifting a small section on one side of the landscape to the alternative stable state.

We used the *pdepe* solver in Matlab to solve the partial differential equations [34]. Boundaries were defined to be reflective. To check that our results were not influenced by the spatial discretization method used in *pdepe*, we repeated all simulations for 100, 1000 and 5000 grid cells.

## Results

**Fig 2:**
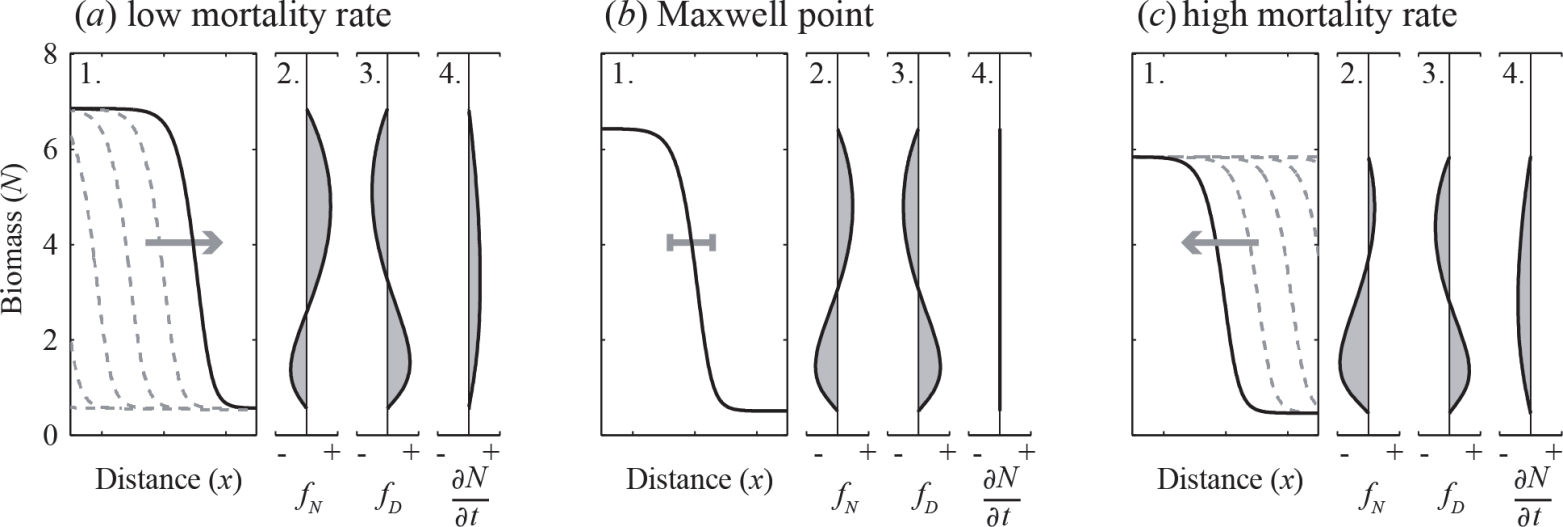
Simulations in a homogeneous landscape with local alternative states and diffusion of the modeled species. Initially, the left side of the landscape is set to the high biomass state, and the right side to the low biomass state. With these initial conditions a moving front establishes, shifting the entire landscape to the state with the highest resilience (*a*) *c* = 2.2, (*b*) *c* = 2.3487, (*c*) *c* = 2.5 (g m^−1^ d^−1^). The four figures in each panel represent: 1) snapshots of the moving front with the grey arrows indicating the shifting direction (scale = 30 m), 2) the local change in biomass per day due to growth and mortality (*f*_*N*_), 3) the local change in biomass per day due to diffusion (*f*_*D*_), and 4) the net local change in biomass per day$\left(\frac{\partial N}{\partial t}=f_{N}+f_{D}\right)$. Note that local dynamics and diffusion precisely cancel out if conditions are such that the modeled system is at the Maxwell point (panel *b*). The scale of all change-in-biomass plots ranges from -0.5 to 0.5 (g m^−1^ d^−1^).

We first show how the resilience of alternative stable states in homogeneous spatially extended systems differs from the classical well-mixed systems. At first, we initialized the landscape such that the population was in the high biomass state on one half of the landscape, and in the low biomass state on the other half. On either side of the created boundary the local dynamics driven by diffusion (*f*_*D*_) have an equalizing effect, while the growth and mortality dynamics (*f*_*N*_) drive the system back to each equilibrium state. The net effect around the front $\left(\frac{\partial N}{\partial t}\right)$ drives the system locally towards one of the states (Fig. 2). This shift propagates further through the landscape resembling a domino effect. Such moving front is called a ‘travelling wave’ [7].

Assuming other conditions to be constant, the maximal mortality rate *c* determines the direction of the travelling front. If the maximal mortality rate is low, a front of high biomass propagates through the landscape, leaving the entire system in the high biomass state (Fig. 2*A*). If the maximal mortality rate is high, the low biomass state is the one that propagates through the landscape (Fig. 2*C*).

There is a single critical value of the mortality rate at which the resilience of both states is equal. At this specific parameter setting, the wave speed is zero (Fig. 2*B*). This point is called the Maxwell point [7,35], where both states have the same potential energy, and therefore the base level in a stability landscape should be the same for both basins of attraction [36]. At the Maxwell point, the dynamics driven by local growth and mortality exactly compensate for the local dynamics driven by diffusion (Fig. 2*B*, S2 Text, S1 Fig.). For single-variable models with local alternative stable states, the set of conditions for the Maxwell point can be mathematically derived from the growth function (S2 Text) [35,37,38], and is generic for any single-variable model with local alternative stable states and diffusion (S2 *A-S2B Fig.*).

The key result is that if either side of the landscape is in one alternative state, a travelling wave always leaves the system in the state with the highest resilience (or more precisely in the state with the lowest potential energy). As a result, in such a simple system we have described, alternative stable states cannot co-exist in a homogeneous landscape. In our single-variable model, the conditions for the Maxwell point are independent of the diffusion rate. However, in a multi-variable model, such as the well-known macrophyte-turbidity model representing shallow lakes dynamics (S2 *E-S2F Fig.*), the rates of exchange of each variable can be different, which has an effect on the stability of either state. For instance in the shallow lakes example, the macrophyte-dominated state will become more resilient against local perturbations if the dispersal rate of macrophytes increases (S3 Fig.). Most importantly however, for multi-variable models with local alternative stable states and random dispersal of key species or diffusion of nutrients, the same key result holds.

On an ‘infinitely’ sized landscape, the most resilient state is thus ‘indestructible’, in the sense that no local disturbance, independent of the size of the disturbance, is able to propagate through space, because conditions are such that a travelling wave in the direction of dominance of the least resilient state cannot occur (Fig. 2). However, if conditions change, such that the system crosses the Maxwell point, the system becomes vulnerable to local disturbances, ending this indestructibility. Environmental conditions will thus determine whether a strong local disturbance in a spatial system can potentially propagate or not.

**Fig 3:**
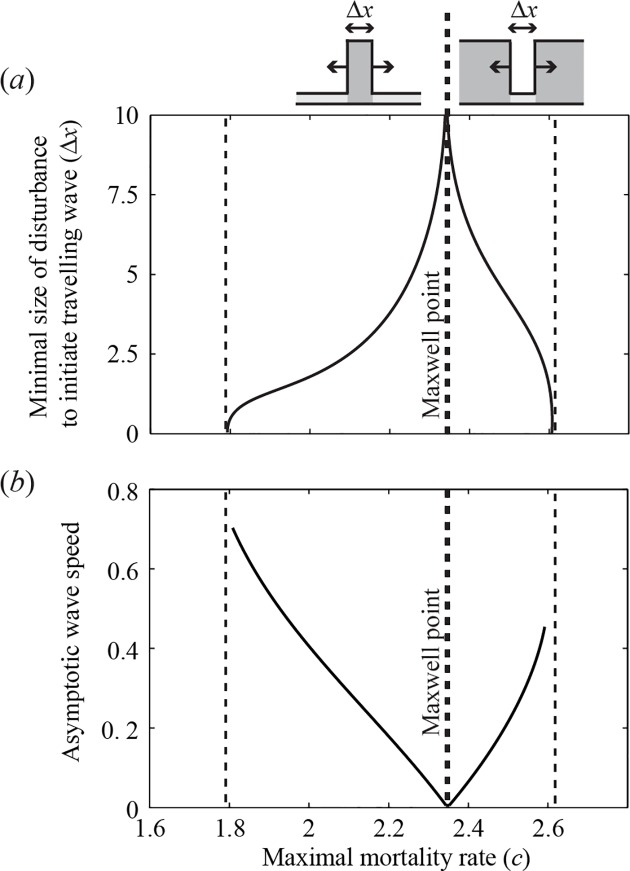
Critical size of a local disturbance and the speed of a travelling wave as a function of the maximal mortality rate *c*. *(a)* On an infinitely sized landscape, disturbances smaller than the critical size *Δx* (in m) are repaired, while larger disturbances will initiate a propagating wave that travels through the landscape with *(b)* a constant wave speed (in m d^−1^). The thick dashed line represents the Maxwell point. The thin dashed lines represent the two fold bifurcations in a non-spatial system. Left of the Maxwell point the entire landscape was initially set to the low biomass state, and the disturbance was set to the high biomass state. Right of the Maxwell point the landscape was initially set to the high biomass state, and the disturbance was set to the low biomass state (indicated by the small upper panels). In this model, an *n*-fold increase in diffusion rate leads to a $\sqrt{n}$-fold increase in both critical disturbance size and wave speed.

Moreover, for a travelling wave to develop, a critical area needs to be disturbed to the alternative state. The actual critical size of this disturbed area (Δ*x*) increases towards the Maxwell point (Fig. 3A). The asymptotic speed of a travelling wave between alternative states is constant on a homogeneous landscape [39], and approaches zero towards the Maxwell point (Fig. 3B). A non-dimensional version of our model (see S1 Text) shows that an *n*-fold increase in diffusion rate *D* leads to a $\sqrt{n}$-fold increase in both *x* and wave speed (see S4 Fig.). Importantly, once the system has shifted to the alternative state, a wave travelling backwards cannot establish, so the new state is on its turn indestructible against local disturbances to the other state. Note that if a system is indestructible against local disturbances, such as fires or storms, it is not indestructible against disturbances that affect the entire landscape, such as periods of drought.

**Fig 4:**
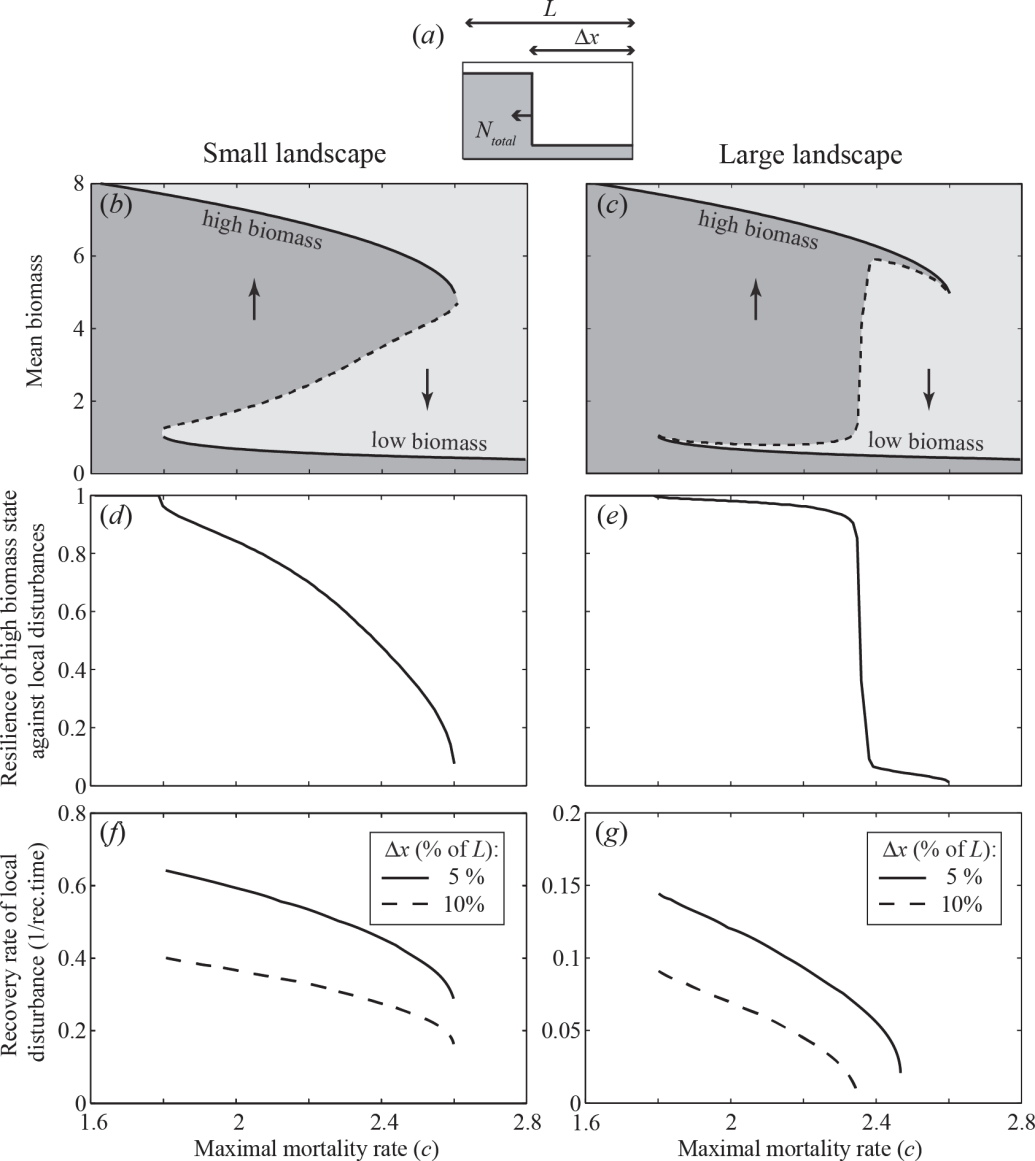
Resilience to local disturbances on a small and a large landscape. (*a*) Local disturbances to the alternative stable state (i.e. the low biomass state) were performed on one side of the landscape, in order to have a symmetrical landscape. (*b*) Mean biomass on the landscape in equilibrium (*N*_*total*_/*L*) as a function of the maximal mortality rate for a system on a relatively small landscape (*L* = 5 m). The solid parts of the curve represent the two stable landscape-wide equilibria. The dashed part of the curve represents the disturbance threshold, i.e. the size of the disturbed patch needed to induce a systemic shift to the alternative stable landscape-wide state. (*c*) The same as panel *b* but for a system on a large landscape (*L* = 50 m). Note that the disturbance threshold remains very close to the less resilient of the two stable equilibria implying that only a small disturbance is needed to induce a shift to the more resilient landscape-wide state. (*d*) Resilience of the high biomass state, in terms of the fraction of the landscape that needs to be perturbed to the alternative state to trigger a shift (Δ*x/L*), for a system on a small landscape. (*e*) The same as panel *d* but for a system on a large landscape. Note that resilience shows a steep drop. (*f*) Engineering resilience of the high biomass state, in terms of the recovery rate of a local disturbance to the low biomass state for a system on a small landscape. (*g*) The same as panel *f*, but for a system on a large landscape.

So far, we considered a landscape without borders, to rule out any edge effects. We now turn to the more realistic case of finite landscapes. In case the landscape is small (or diffusion is high, which is mathematically the same) the system does in practice behave like a well-mixed system (Fig. 4*B*). Consequently, shifts will occur almost simultaneously across the landscape. In contrast, if the landscape is large, a local disturbance can cause a travelling wave that propagates through space, always in the direction of the least stable state. A relatively small disturbance can therefore already lead to systemic collapse (Fig. 4*C*). As a result, resilience, here defined as the capacity to recover upon a local disturbance, of the alternative states changes abruptly around the Maxwell point (Fig. 4*E*, see S2 Fig. for other models). As stress on the dominant state increases (e.g. through the mortality rate in our model), resilience to local disturbances remains unaltered in the sense that the system will recover, even from large disturbances, until a critical point is reached (the Maxwell point) where resilience sharply drops to a point where even a local disturbance can induce a traveling wave that will eventually bring the entire landscape in the alternative state. This is quite unlike the gradual decrease of resilience on a small landscape (Fig. 4*D*), corresponding to the classical well-mixed situation.

In contrast to the resilience as defined by Holling [1], the recovery rate of a local disturbance (i.e. ‘engineering resilience’) does decrease gradually in a spatially extended system (Fig. 4*G*). In that case, recovery from a local disturbance slows down if conditions are close to the Maxwell point. The recovery rate is slowest just before the point at which the disturbance will trigger a travelling wave of expansion towards the alternative state (Fig. 4*G*). While this point lies beyond the Maxwell point for small disturbances, for large disturbances a wave will be triggered as soon as the Maxwell point is crossed. Importantly, the same key result holds: once a spatially extended system has shifted to a degraded state, it is extremely difficult to re-establish the original desired state without changing global conditions. For example, in the scenario with a large landscape in Fig. 4, one needs to bring more than 90% of the landscape back into the desired state; otherwise the remaining degraded patch will, although slowly, expand again (Fig. 4E).

In all scenarios we examined so far, the spatially extended system eventually ends up in one state. Situations in which the alternative states occur side-by-side in a homogeneous landscape can be induced by strong local perturbations, but are transient. For stable co-existence of alternative stable states we need to relax the assumption that the environmental conditions determining growth and mortality parameters as well as diffusion rates are entirely homogeneous in space.

**Fig 5:**
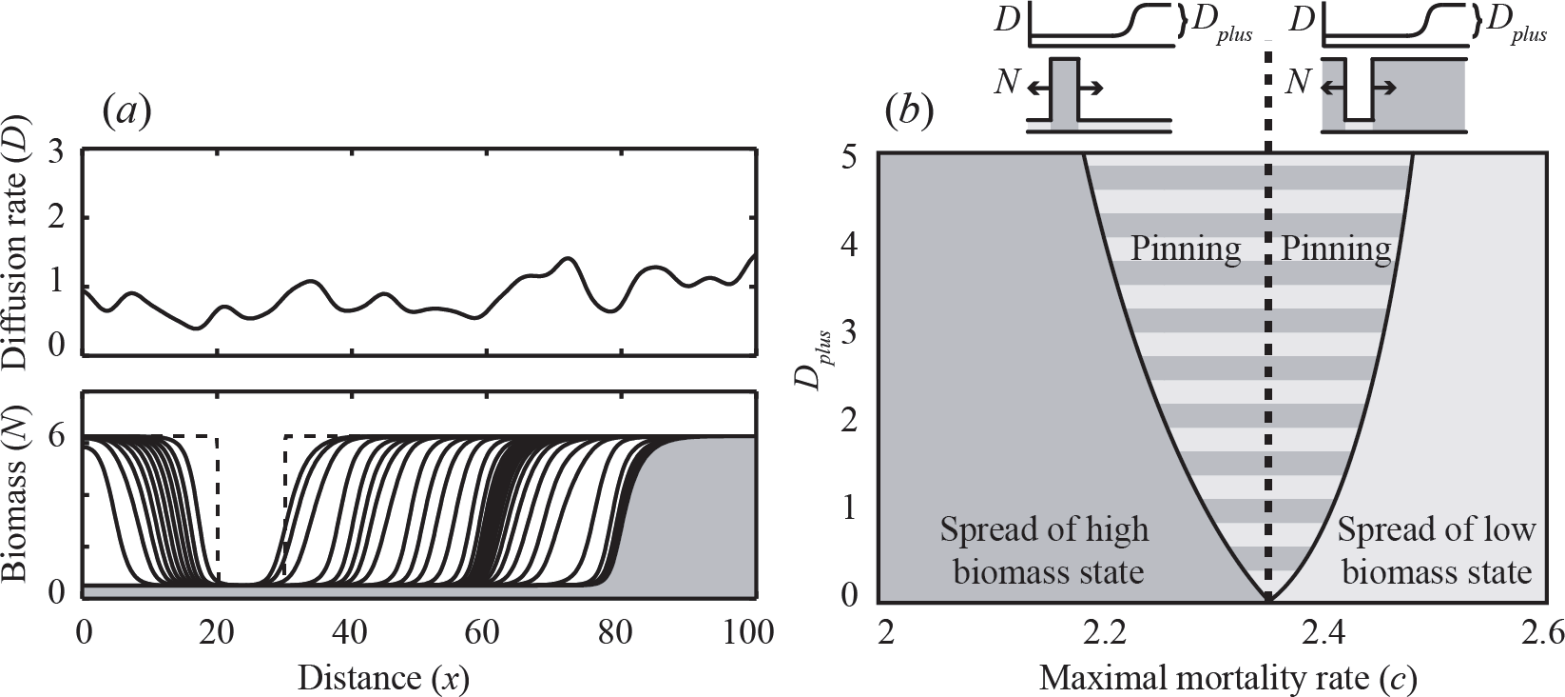
The effect of spatially heterogeneous diffusion. A travelling wave of collapsing biomass triggered by a disturbance can come to a halt if it meets an area of increased diffusion rates. (*a*) The effect is illustrated in a simulated landscape with heterogeneous diffusion rates (*c* = 2.4 g m^−1^ d^−1^) (upper panel). The dashed line in the lower panel represents the initial disturbance and the solid lines depict the transient situation every 40 days. The shaded area depicts the final stable configuration. This configuration is stable, as long as the system does not suffer from other local disturbances. (*b*) In order to understand the conditions for pinning, we introduced a local disturbance in a landscape with a single spatial gradient in diffusion rate, representing a change from an area with low diffusion (*D*_*0*_) to an area with high diffusion (*D*_*0*_+ *D*_*plus*_) (visualized in the small upper panels). The landscape was created by a sigmoidal function: $f_{D}(N,x)=\frac{\partial }{\partial x}\left(\left(D_{0}+D_{plus}\frac{x^{p}}{x^{p}+{\left(L/2\right)}^{p}}\right)\frac{\partial N}{\partial x}\right)$(*D*_*0*_ = 1 m^2^d^−1^_,_ *p* = 50, *L* = 100 m). The main panel represents the occurrence of pinning for different combinations of maximal mortality rate *c* and the level of increase in diffusion rate *D*_*plus*_. Importantly, pinning only occurs if a traveling wave meets an area in which diffusion is higher. The thick black dashed line indicates the Maxwell point.

We first simulated landscapes with spatially variable diffusion rates (Fig. 5). This can be seen as an intermediate between two simplified spatial models: a spatially discrete model with exchange between patches and a continuous model with homogeneous diffusion (see S3 Text and S5 Fig.). The results show that a travelling wave can slow down and can come to a halt, if it meets an area of *increasing* diffusion rates (Fig. 5*A*). Whether a travelling wave comes to a halt, so-called ‘pinning’, depends on three factors. Pinning is more likely if i) the actual level of increase in diffusion (*D*_*plus*_) is high (Fig. 5*B*), ii) the scale on which the increase in diffusion occurs is low, i.e. the steepness of the diffusion function (set by *p* in the sigmoidal function described in the caption of Fig. 5) is high (S3 Text, S6 Fig.), and iii) there is little difference between the resilience of the alternative states, causing the system to be close to the Maxwell point (Fig. 5*B*). Thus even if growth and mortality rates are homogeneous, spatial variation in exchange or dispersal rates can allow spatial co-existence of alternative stable states.

**Fig 6:**
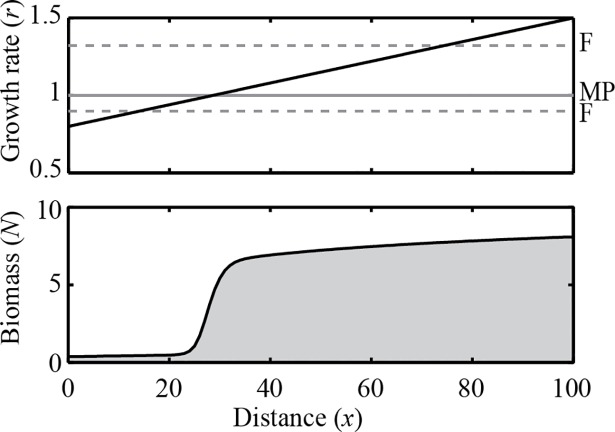
The effect of spatially heterogeneous conditions. A gradual increase in growth conditions in space (e.g. north-south gradient in temperature) results in a distinct shift in space from the low biomass state to the high biomass state (i.e. a stable standing wave) on the location where conditions cross the Maxwell point. Upper panel: The solid black line represents the local growth rate *r* on the landscape. The solid grey line indicates where the conditions cross the Maxwell point (MP), and the two dashed grey lines indicate where the two fold bifurcations (F) are crossed. Lower panel: The shaded area represents the stable end configuration of biomass for any initial configuration (*c* = 2.35 g m^−1^ d^−1^).

Next, we simulated heterogeneity in environmental conditions in the most straightforward manner, using a spatial gradient of growth rate *r* (Fig. 6). One can, for example, imagine a gradient in water availability related to distance to water source, or precipitation and temperature gradients related to latitude. With a smooth spatial gradient in the growth rate, a sharp boundary between two states is formed simply wherever conditions cross the Maxwell point. If both sides of the landscape are connected to an area in which the system has only one stable state, this configuration is completely independent of initial conditions (Fig. 6). As a consequence, as long as diffusion rates are homogeneous, and there is a smooth gradient in environmental conditions, there will be no local nor large-scale hysteresis effects in the response to a changing environmental variable. If global conditions change gradually, the location of the spatial shift will also shift in a gradual manner.

In natural systems, however, one would expect environmental conditions to be more heterogeneous than along a simple gradient. In that situation, a slow, gradual change in global conditions would likely lead to incidental ‘local waves’ of change (see S4 Text, and S7 Fig.). These local waves would cause a reverse change if global conditions are reversed. In contrast, if exchange or dispersal rates are heterogeneous is space, a slow, gradual reversal in conditions would not simply cause a reverse change in the landscape as a local increase in exchange or dispersal of a key variable in one direction would not lead to ‘pinning’ in the other direction (Fig. 5).

## Discussion

Our results illustrate that even for entirely homogeneous landscapes, stability properties of a spatially extended ecosystem can differ profoundly from those of a simple, well-mixed ecosystem. Differences are especially intriguing when it comes to ecological resilience, generally defined as the maximum disturbance that a system can tolerate without switching to the alternative stable state [1]. Here, we specify this definition to local disturbances, and define ecological resilience as the size of the area of a strong local disturbance needed to trigger a shift. If the ecosystem is spatially extended, the least resilient state tends to be always fragile (Fig. 4*C*) in the sense that a strong local disturbance can already trigger a domino effect in the form of a travelling wave (Fig. 2) that leaves the landscape in the alternative more resilient state. Importantly, as environmental conditions change, there is a sudden drop in resilience at the point where one state becomes more resilient than the other (i.e. at the Maxwell point) (Fig. 4*E*), rather than the classical gradual decrease towards the fold bifurcation (Fig. 4*D*). In fact, this class of systems may hardly have hysteresis in practice as only a small patch needs to be in the most stable state to trigger a system wide shift, and the new state is stable against local disturbances (Fig. 4*C*). Mathematically, these results on travelling waves and the Maxwell point are not new [7], and have been discussed in the context of populations with an Allee effect [11–13]. However, surprisingly, the connection to the concepts of resilience and hysteresis has not been made so far.

Effectively, a spatially extended system in our model means that a positive feedback acts on a small scale relative to the size of the landscape, which is often the case in ecosystems. For example, foundation species such as *Spartina* (cordgrass) on intertidal flats facilitate vegetation development, by locally lowering erosion levels [40]. Seagrass facilitates neighboring seagrass development by trapping sediment leading to increased light conditions [41] and decreased erosion levels, but it will depend on the water mixing rate how far this effect can reach. Similarly, but on a much larger scale, local development of forest suppresses wildfire propagation [42], but has no effect on fires at a longer distance. Our results suggest that ecosystems that have alternative stable states in small-scale experiments or on a small landscape, could, on a larger landscape, be surprisingly resilient (Fig. 4*E*). This may explain why, after a large-scale die-off of *Spartina* due to drought, snail grazing, and fungal infections [43], remnant *Spartina* patches (if large enough) could slowly expand into die-off areas. Survival is possible, because *Spartina* facilitates expansion locally. On the other hand, once the Maxwell point has passed, the degraded (or undesired) state will also be surprisingly resilient. This could explain the persistent failure of local restorations in certain systems, such as the lack of survival of planted seagrass patches in the Wadden sea where this plant used to dominate [44].

It is important to note that the systems we studied are different from those with self-organized Turing patterns [45]. Such patterns can arise in situations where local positive feedbacks are associated to a depletion of resources or increased stress levels (i.e. erosion) at a distance. For instance, vegetated patches in an arid landscape can locally increase soil permeability, which has a positive effect on local plant growth. However, at a distance from the patch this may lead to water depletion, and thus to unfavorable plant growth conditions [46]. Similarly, mussels or diatoms may locally protect themselves and their neighbors from wave action and erosion, but by doing this they increase these stress levels at a distance [47,48]. The combination of a local positive feedback, and a sufficiently strong long-range negative feedback can, without intrinsic heterogeneity in abiotic conditions, lead to regular spatial patterns (i.e. spots, labyrinth, gaps) [45].

**Fig 7:**
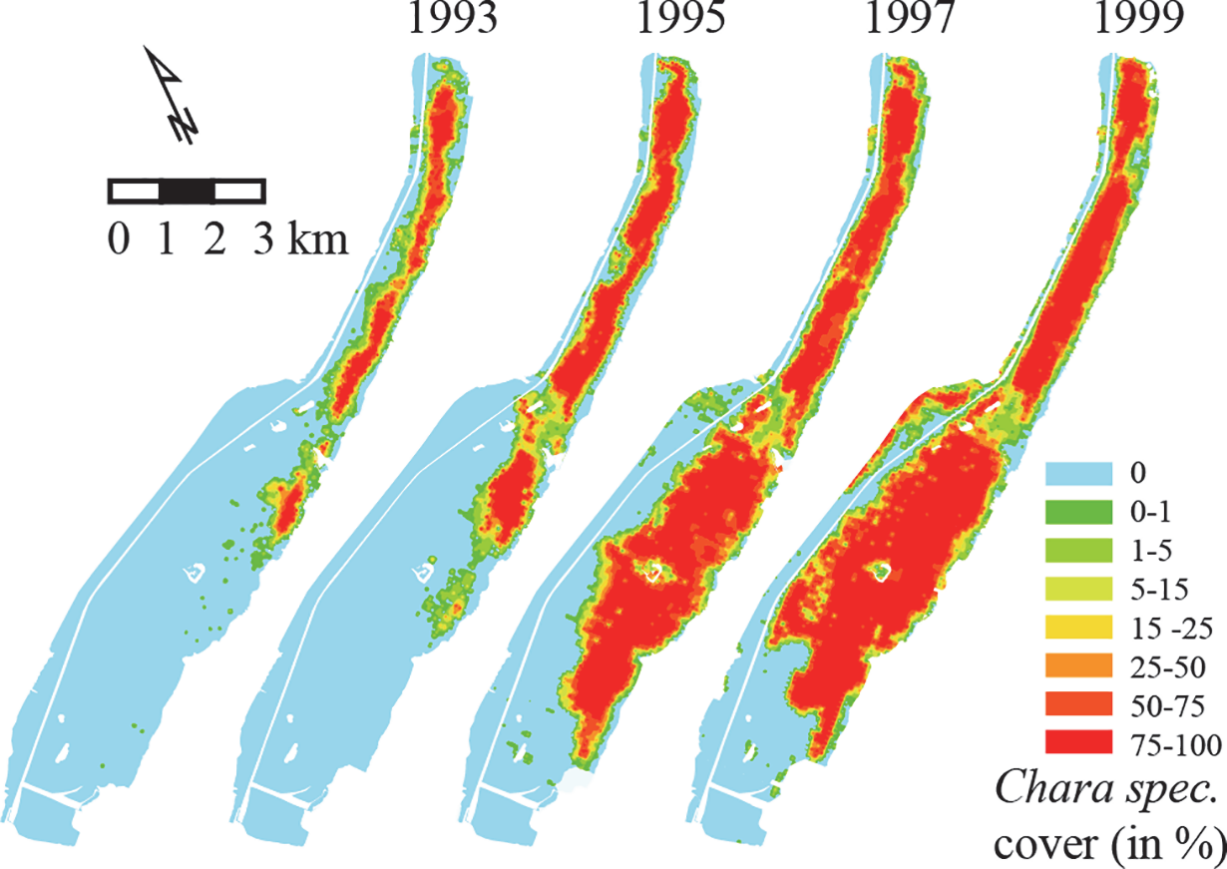
Travelling wave-type of spread of aquatic vegetation (*Chara spec*). Lake Veluwe, the Netherlands, from 1993 to 1999 (from: Monitoring of aquatic vegetation of the IJsselmeer Area by Rijkswaterstaat, an Agency of the Ministry of Infrastructure and the Environment, The Netherlands).

The conspicuously regular patterns of contrasting states in such self-organized systems tend to be very robust. By contrast, our analysis suggests that stable coexistence of alternative states (without a long-range negative feedback) in a homogenous landscape might be unlikely, even though the movement of the front between states can be slow in practice. Indeed, field observations do suggest that some sharp borders between alternative states may represent transient situations rather than stable states. For instance, following improved water quality, patches of clear water with submerged water plants arose during the summer of 1993 in the Dutch lake Veluwe. At that time, this was thought to be an indication of stable co-existence of alternative stable states [18]. However, over the years, these patches of clear water with submerged water plants have gradually spread to a patch as large as almost the entire lake (Fig. 7). Similarly, van Wesenbeeck et al. [19] describe the slow spatial spread of vegetation cover in saltmarsh pioneer zones as being non-stable at longer timescales.

In the context of our model analyses it becomes clear from such observations that the existence of a sharp border between ecosystem states in an otherwise homogeneous landscape should be interpreted with care when it comes to the framework of alternative stable states. Close to the Maxwell point, borders may simply move very slowly (Fig. 4*G*) [7], resulting in apparent co-existence at shorter timescales, even if one state may eventually dominate. It is also important to note that if an ecosystem is subject to occasional stochastic disturbances or recurrent temporal change (e.g. seasons), the slowness of the movement of fronts would allow the existence of a permanent (albeit spatially unstable) patchwork of alternative states [49].

Clearly, situations with sharp borders between alternative states may often occur in a stable way along smooth environmental gradients (Fig. 6). Such situations do in a sense represent critical transitions in space. This is analogous to classical critical transitions in time, except that environmental conditions now change over space rather than over time (e.g. [50,51]). At the established border the positive feedback is too weak to trigger a local shift towards the other state, leading to a dynamic equilibrium. This type of dynamics has, for example, been suggested to be the underlying mechanism for sharp borders of clear vegetated water in a river stretch with increasing background turbidity [26], sharp mussel bed borders on an intertidal flat with increasing wave exposure [21], and northern boreal forest boundaries as a result of latitudinal gradients in temperature [10].

Interestingly, our model analysis also suggests that even if environmental conditions for growth and survival are homogeneous, spatial coexistence of alternative states can still be stable if diffusion rates are not entirely homogeneous through space (Fig. 5 and S6 Fig.). One may think for example of internally mixed lakes that are connected through channels [16], or natural areas fragmented by roads or other landscape elements that limit dispersal of species. The mechanism behind this particular case of co-existence of alternative states is that a local increase in exchange rates causes the area from which each of the states draw for additional inputs (the ‘hinterland’) to vary from place to place, thus effectively causing the strength of the positive feedback to vary over space. As a consequence, expansion can be halted allowing stable co-existence of the low and high biomass state (Fig. 5). From a modeling point of view, this result demonstrates that stable co-occurrence of alternative stable states in space is not an artifact of using discrete space models [11] (see S5 Fig.). Rather, the fact that such co-existence is impossible in idealized continuous space models may be seen as an artifact of neglecting the possibility of heterogeneity in dispersal rates. Obviously, animals and plants can have more complex dispersal strategies than simple random dispersal. The potential for co-existing alternative stable states is expected to be lower with long-distance seed dispersal, or active dispersal towards unoccupied areas, since dispersal barriers can be crossed more easily.

### Implications

Clearly it is challenging to bridge the gap between our fundamental understanding of alternative stable states on small scales, and the dynamics of spatially extended ecosystems. Our results are limited to basic principles derived from simple models. Nonetheless, they help to see why scale can prohibit restoration of spatially extended homogeneous ecosystems such as estuaries or very large lakes. Petraitis and Latham [52] already hypothesized that ‘the spatial and temporal window of opportunity of a perturbation must be large enough to gain a foothold and initiate pivotal positive feedback processes’. Indeed, our model predictions are in line with findings that local restoration efforts must be large enough to gain momentum for a travelling wave to establish. On the other hand, our results also imply that even if the desired state is stable in an isolated system (e.g. mesocosms or enclosures) it may not be able to spread through space.

In addition to providing a framework for understanding resilience and restoration of spatially extended systems, our results suggest mechanisms leading to entirely different ways in which major biomes might reorganize in response to climate change. Tropical and boreal forests are the two largest biomes on Earth. As climate changes, the regions dominated by these two types of forest will likely change, but the character of the transitions is unclear. Recent studies suggest that, under certain conditions, tropical forest and savanna are alternative stable states [9,53] and the same may be true for boreal forest and tundra, and tundra and steppe [10]. It has been suggested that more or less stochastic local switches may give the range shift of tropical forest an overall gradual character [8]. Our results suggest that this scenario is indeed likely if environmental heterogeneity follows a smooth gradient (e.g. temperature gradient related to latitude), as in Fig. 6. On the other hand, one could speculate that over large homogeneous areas, a massive shift to an alternative biome might occur once a tipping point is reached (see S4 Text and S7 Fig.). If we assume that currently homogeneous systems signal a tendency to a winner-takes-all situation, our results suggest that such biomes might be likely to shift to an alternative state through massive traveling waves, called ‘gradual regime shifts’ by Bel et al. [7], to differentiate them from abrupt landscape-wide shifts. However, the speed of such waves would depend among other things upon the rate at which states disperse. For example, the spread of boreal trees into the tundra may be limited by seed dispersal [54]. On the other hand, the rate of disappearance of boreal forest at the Southern end of its range as temperatures rise could be dictated by massive wildfires and insect outbreaks as already observed [55].

## Conclusion

Our analysis illustrates that the resilience of spatially extended ecosystems will differ markedly from those of small isolated parts of such systems. Even if a positive feedback causes a system to have alternative stable states on small scales over a range of conditions, this hysteresis will tend to disappear in a larger spatial context. This is because in a spatially extended system one of both alternative states will tend to be dominant in the sense that it is highly resilient against local disturbances. By contrast, in the alternative (subdominant) state, even a small local disturbance will tend to invoke a shift to the dominant state that spreads through the entire landscape as a travelling wave. Environmental conditions determine the resilience of both the dominant and subdominant state and at the so-called Maxwell point the dominance shifts between the states. Close to this point the travelling wave moves very slow. This allows long transient co-occurrence of alternative states side-by-side, and also implies that even minor spatial barriers can lead to stagnation of a travelling wave and thus to permanent co-existence of alternative states in space.

Obviously model studies as the ones presented in this paper can only hint at potential classes of mechanisms and dynamics. Combinations of field experiments, elaborate models and analyses of field patterns will be required to reduce the uncertainty we have about the mechanisms that rule the large scale dynamics of spatially extended systems. In any case our results illustrate that stability properties on large scales cannot be deduced from small-scale experiments alone.

## Supporting Information

S1 Fig Conditions for the Maxwell point in relation to the growth function. The shape of the growth function determines the direction and speed of the travelling wave. Wave speed is zero when the shaded areas on both sides of the unstable equilibrium have equal size (here at *c* = 2.3487, *MP* = Maxwell point).
(TIF)

S2 Fig The Maxwell point and resilience of three models with local alternative stable states (see S1 Table). (*a*-*b*) eutrophication model with nutrient input *a* as the control parameter; (*c*-*d*) Allee effect model with exploitation rate *f* as the control parameter; and (*e*-*f*) vegetation and light attenuation model with background light attenuation level *E*_*0*_ as the control parameter. The thick dashed line in the left panels indicates the level of the control parameter at the Maxwell point (MP). The right panels show the resilience, in terms of the fraction of the landscape that needs to be perturbed (i.e. with a strong local perturbation) to trigger a shift to the alternative stable state of (*b*) the low nutrient state in the eutrophication model; (*d*) the high biomass state in the Allee effect model; and (*f*) the vegetated, clear state in the vegetation—light attenuation model. For all three models, the size of the landscape (*L*) is 100 m.
(TIF)

S3 Fig The Maxwell point in the vegetation—light attenuation model. (*a*) The range of conditions holding the Maxwell point. The actual conditions for the Maxwell point depend on (*b*) the ratio between the dispersal rate of vegetation and the mixing rate of turbidity (*D*_*V*_ /*D*_*E*_). Note that the location of the Maxwell point influences the resilience of the two global states against local perturbations.
(TIF)

S4 Fig Critical size of disturbance and wave speed as a function of the re-scaled mortality rate σ. (*a*) Critical size of a local disturbance as a function of the re-scaled maximal mortality rate *σ*. Disturbances smaller than the critical size are repaired, while larger disturbances propagate through the landscape, shifting the entire landscape to the alternative state. The thick dashed line represents the Maxwell point. Left of the Maxwell point the entire landscape was initially set to the low biomass state, and the disturbance was set to the high biomass state. Right of the Maxwell point the landscape was initially set to the high biomass state, and the disturbance was set to the low biomass state (indicated by the small upper panels). (*b*) Mean biomass on the landscape in equilibrium (*N* ^*^_total_/*L* ^*^) as a function of the re-scaled maximal mortality rate for two systems with landscape size *L* ^*^. The solid parts of the curve represent the two stable landscape-wide equilibria. The dashed parts of the curve represent the disturbance thresholds, i.e. the size of the disturbed patch needed to induce a systemic shift to the alternative stable landscape-wide state.
(TIF)

S5 Fig Comparison of a spatially discrete and a spatially continuous model with local alternative stable states. Stable end configurations of the entire landscape in the high biomass state, the entire landscape in the low biomass state, or stable co-existence of both alternative states in space, as a function of the maximal mortality rate and the dispersal rate of species *N*. The initial landscape was set at one half in either of the alternative states in: (*a*) a one-dimensional lattice with discrete grid cells; and (*b*) a homogeneous, spatially continuous landscape. Note that alternative states can co-occur in space on a landscape with patches (*a*), while in a spatially continuous system, a travelling wave will always move the entire system to one of the states (*b*) (main text). The example simulations below are space-time-plots for *c* = 2.2 and *c* = 2.4 (*D* = 10).
(TIF)

S6 Fig The effect of spatially heterogeneous diffusion. A travelling wave of collapsing biomass triggered by a disturbance can come to a halt if it meets an area of increased diffusion (*D*_*plus*_). The probability of this so-called pinning increases if the shift in diffusion becomes less gradual, thus if the steepness of the shift (*p*) increases. Maximal mortality rate: (*a*) *c* = 2.2; (*b*) *c* = 2.3; (*c*) *c* = 2.4 (*d*) *c* = 2.45. As shown by Fig. 5 (main text), the likelihood of pinning is high if the maximal mortality rate is close to the Maxwell point (e.g. panels (*b*) and (*c*)).
(TIF)

S7 Fig The effect of spatially heterogeneous environmental conditions. Example simulations of recovery, following a gradual decrease in maximal mortality rate on a landscape with local alternative stable states in: (*a*) a generally homogeneous landscape with one edge at which the growth rate is locally high (a refuge); (*b*) a landscape with a linear gradient in growth rate; and (*c*) and (*d*) two landscapes with random heterogeneity in growth rate. The thick black line in the lower panels represents the local growth rate *r*. The maximal mortality rate is changed from 2.36 to 1.96, and the simulation results are depicted for steps of 0.02. The stable end configurations of biomass are depicted in the upper panels as shaded areas, ranging from light gray to black. For each parameter setting, the grey solid lines in the lower panels represent the upper fold bifurcation, and the grey dashed lines the Maxwell point. When the growth rate at a point in space exceeds the fold bifurcation, there is locally only one stable state. When the growth rate locally exceeds the Maxwell point, a travelling wave towards the higher biomass could be triggered, provided that the area that initiates the wave is sufficiently large. Note that in an environment with an environmental gradient (panel *b*), the location of a standing wave directly follows changes in global conditions (no hysteresis), while in an environment with random heterogeneity, the location of the standing wave changes stepwise, which can result in hysteresis if the maximal mortality rate decreases again.
(TIF)

S1 Table Model equations and parameters of three models with alternative stable states. (DOCX)

S1 Text The Maxwell point in a range of bistable models. (DOCX)

S2 Text Non-dimensional model. (DOCX)

S3 Text Co-existence of alternative stable states. (PDF)

S4 Text Gradual shifts in heterogeneous spatially extended systems. (DOCX)
