# Supplementary figures and images for: Resilience of Alternative States in Spatially Extended Ecosystems
Source: PLoS One. 2015 Feb 25;10(2):e0116859. doi: 10.1371/journal.pone.0116859 (PMC4340810; doi:10.1371/journal.pone.0116859)

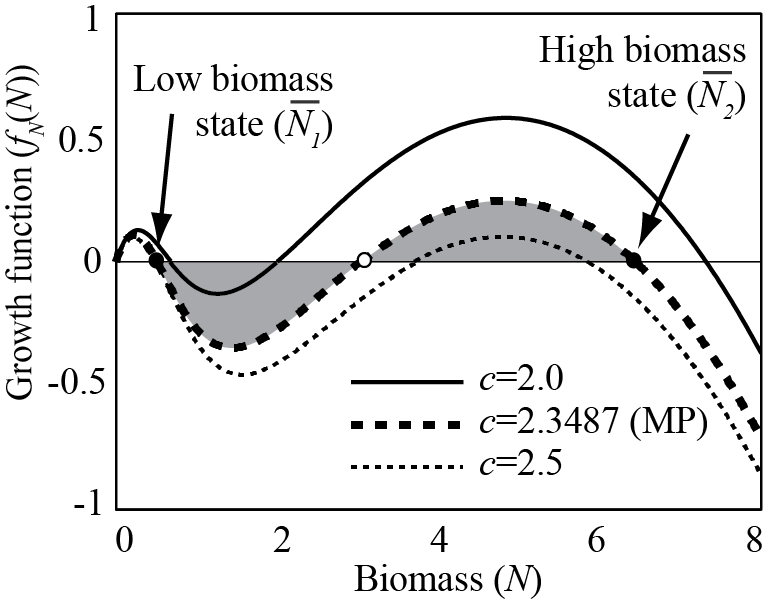

Supplement: S1 Fig — The shape of the growth function determines the direction and speed of the travelling wave. Wave speed is zero when the shaded areas on both sides of the unstable equilibrium have equal size (here at c = 2.3487, MP = Maxwell point). (TIF) [file pone.0116859.s001.tif]

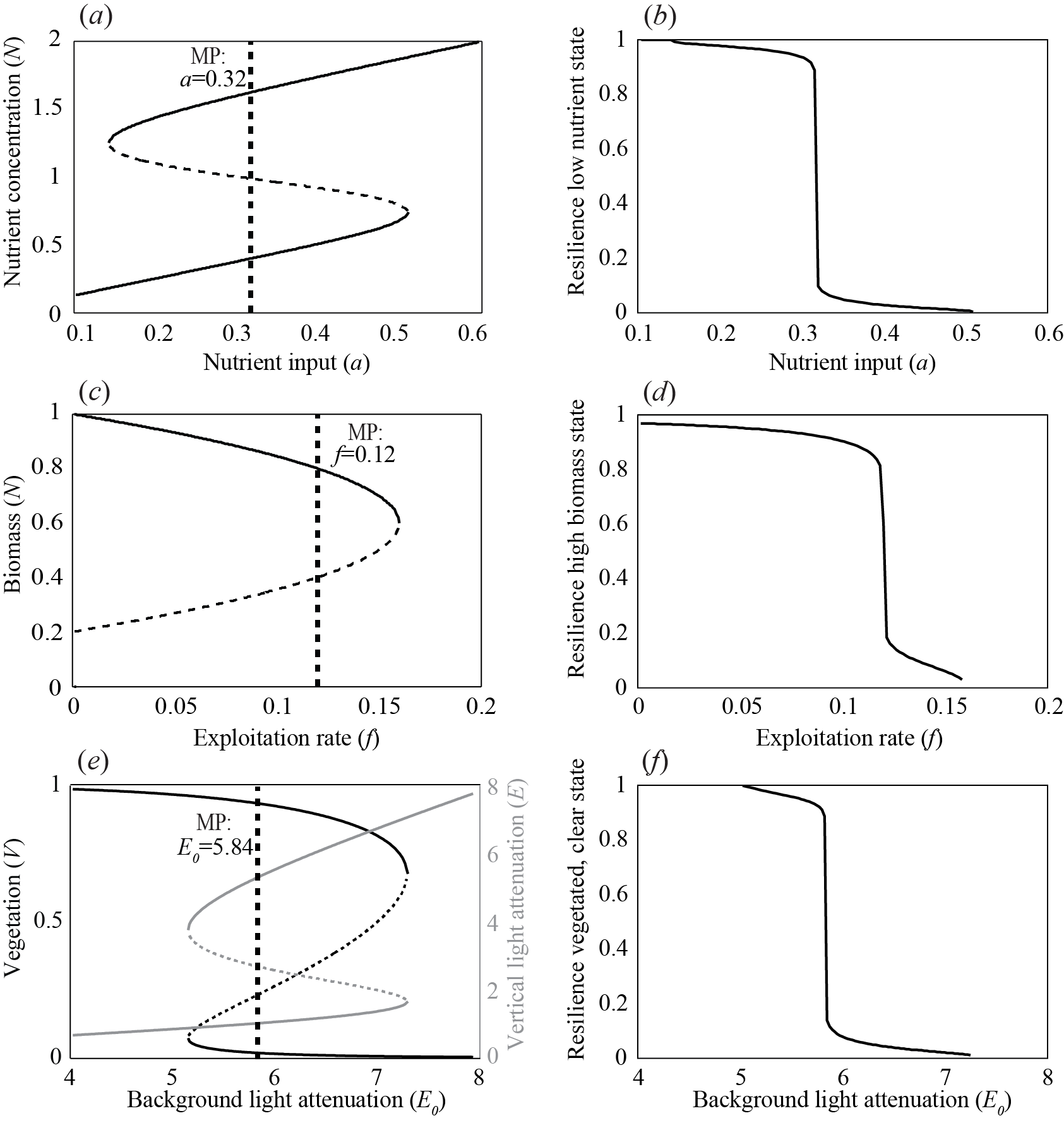

Supplement: S2 Fig — (a-b) eutrophication model with nutrient input a as the control parameter; (c-d) Allee effect model with exploitation rate f as the control parameter; and (e-f) vegetation and light attenuation model with background light attenuation level E 0 as the control parameter. The thick dashed line in the left panels indicates the level of the control parameter at the Maxwell point (MP). The right panels show the resilience, in terms of the fraction of the landscape that needs to be perturbed (i.e. with a strong local perturbation) to trigger a shift to the alternative stable state of (b) the low nutrient state in the eutrophication model; (d) the high biomass state in the Allee effect model; and (f) the vegetated, clear state in the vegetation—light attenuation model. For all three models, the size of the landscape (L) is 100 m. (TIF) [file pone.0116859.s002.tif]

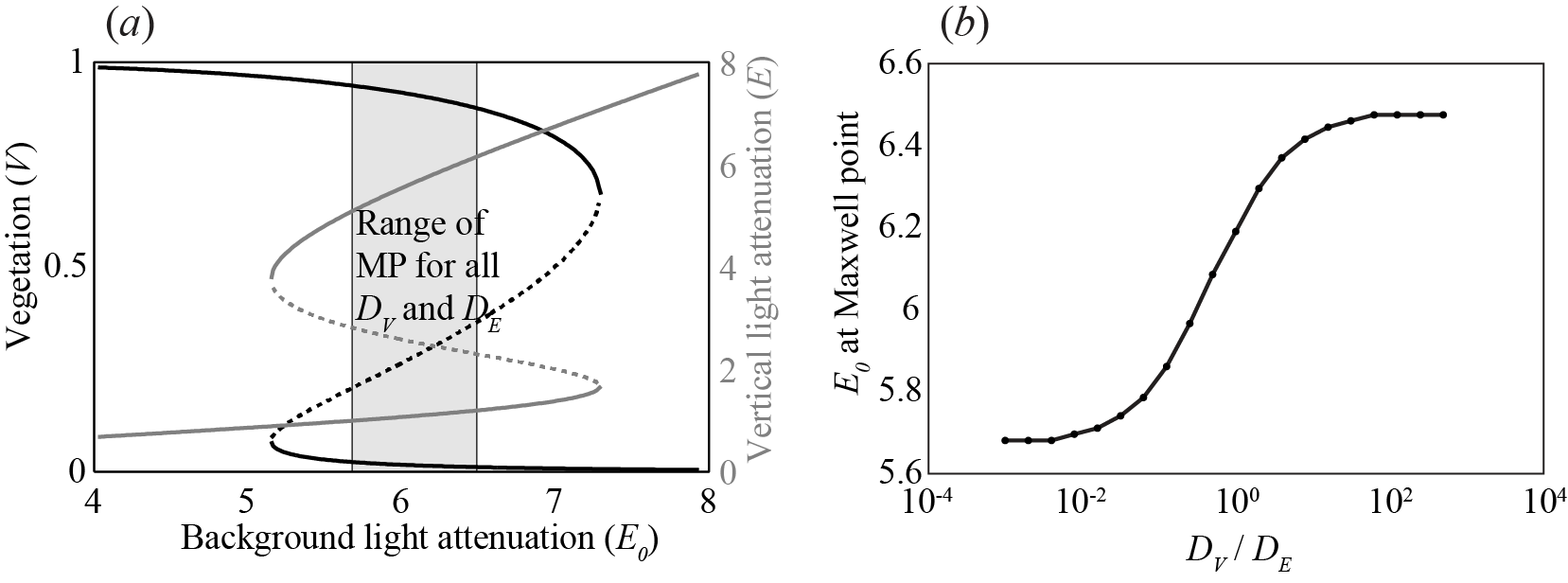

Supplement: S3 Fig — (a) The range of conditions holding the Maxwell point. The actual conditions for the Maxwell point depend on (b) the ratio between the dispersal rate of vegetation and the mixing rate of turbidity (D V /D E). Note that the location of the Maxwell point influences the resilience of the two global states against local perturbations. (TIF) [file pone.0116859.s003.tif]

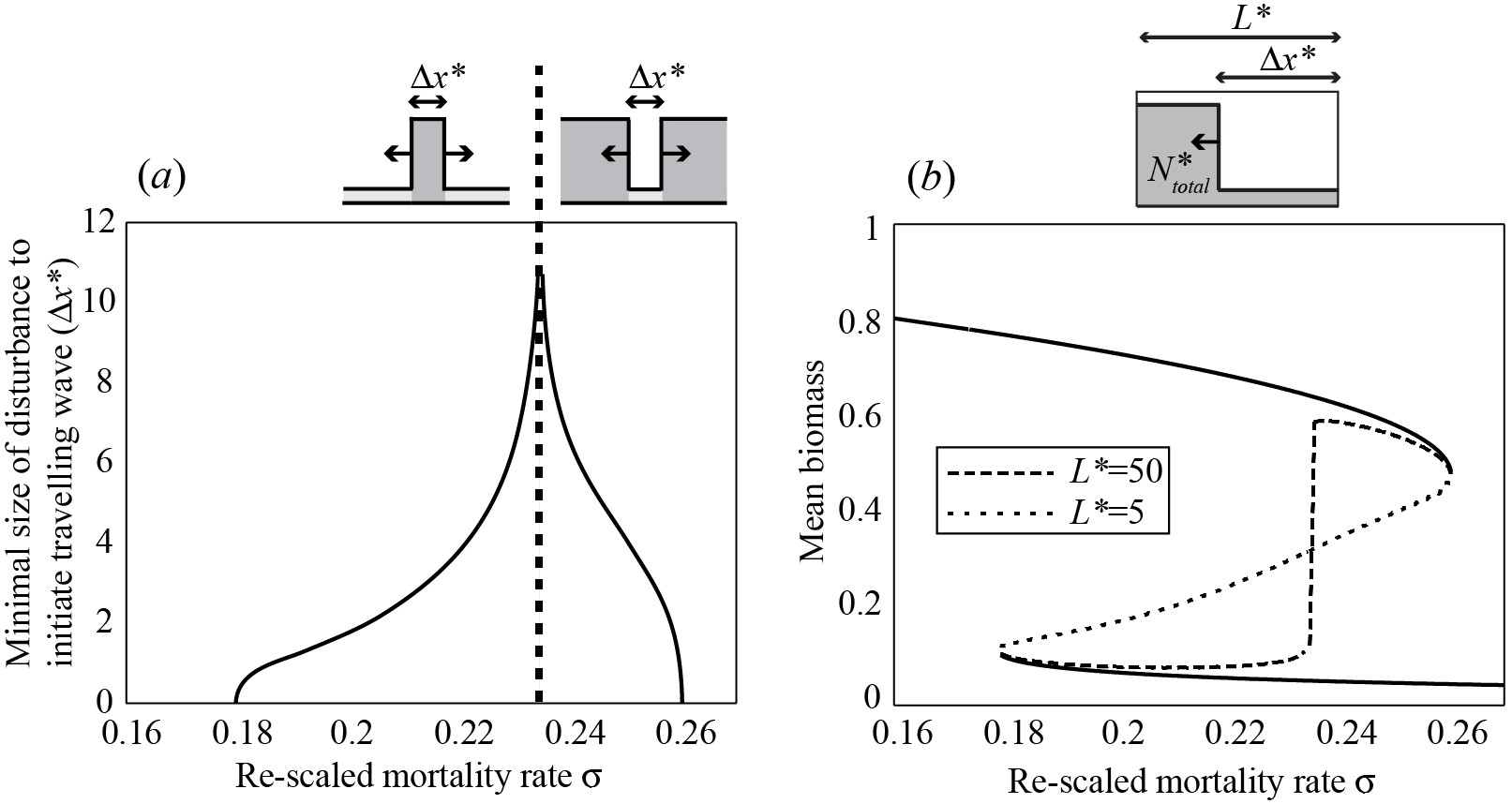

Supplement: S4 Fig — (a) Critical size of a local disturbance as a function of the re-scaled maximal mortality rate σ. Disturbances smaller than the critical size are repaired, while larger disturbances propagate through the landscape, shifting the entire landscape to the alternative state. The thick dashed line represents the Maxwell point. Left of the Maxwell point the entire landscape was initially set to the low biomass state, and the disturbance was set to the high biomass state. Right of the Maxwell point the landscape was initially set to the high biomass state, and the disturbance was set to the low biomass state (indicated by the small upper panels). (b) Mean biomass on the landscape in equilibrium (N * total/L *) as a function of the re-scaled maximal mortality rate for two systems with landscape size L *. The solid parts of the curve represent the two stable landscape-wide equilibria. The dashed parts of the curve represent the disturbance thresholds, i.e. the size of the disturbed patch needed to induce a systemic shift to the alternative stable landscape-wide state. (TIF) [file pone.0116859.s004.tif]

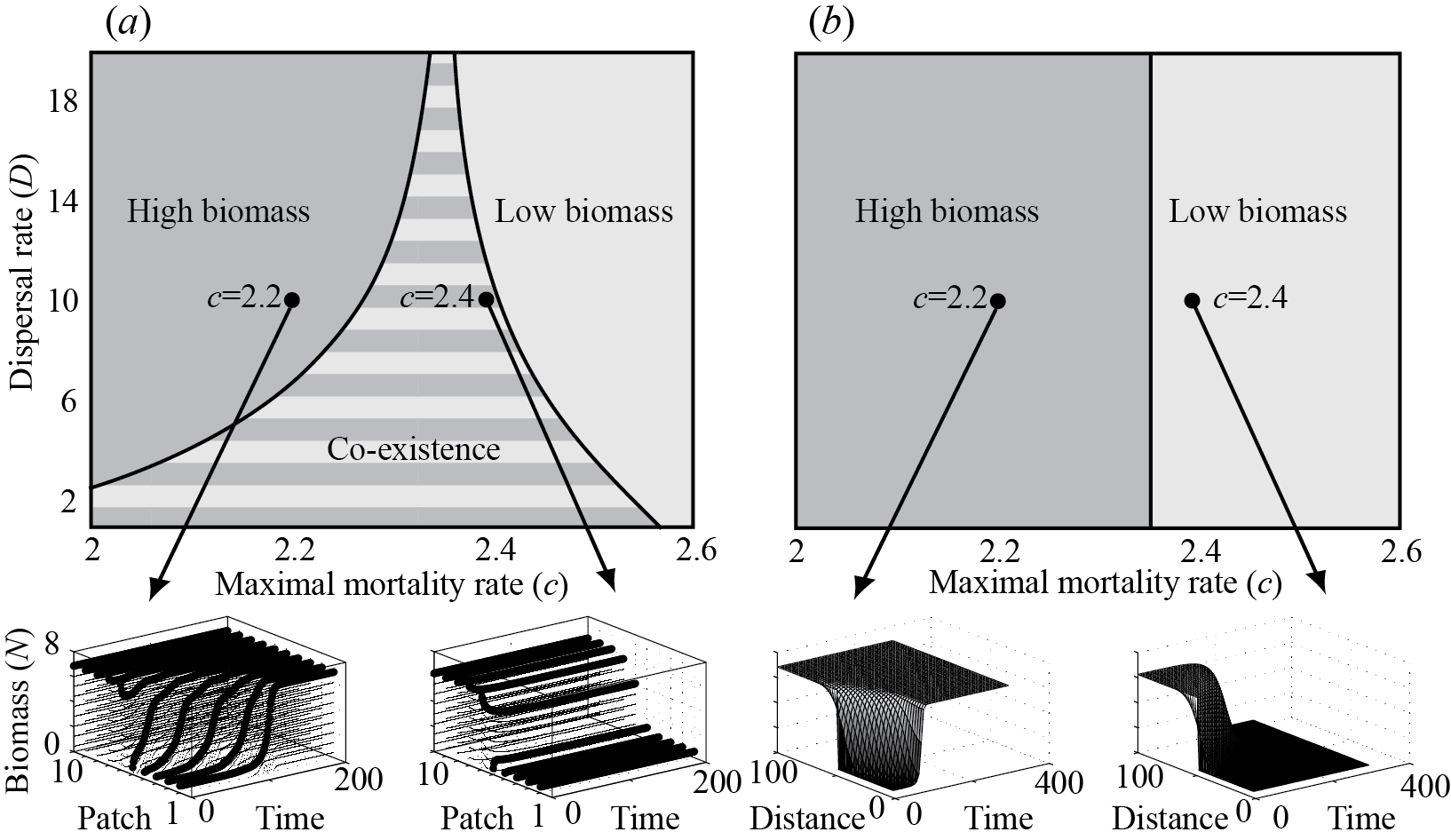

Supplement: S5 Fig — Stable end configurations of the entire landscape in the high biomass state, the entire landscape in the low biomass state, or stable co-existence of both alternative states in space, as a function of the maximal mortality rate and the dispersal rate of species N. The initial landscape was set at one half in either of the alternative states in: (a) a one-dimensional lattice with discrete grid cells; and (b) a homogeneous, spatially continuous landscape. Note that alternative states can co-occur in space on a landscape with patches (a), while in a spatially continuous system, a travelling wave will always move the entire system to one of the states (b) (main text). The example simulations below are space-time-plots for c = 2.2 and c = 2.4 (D = 10). (TIF) [file pone.0116859.s005.tif]

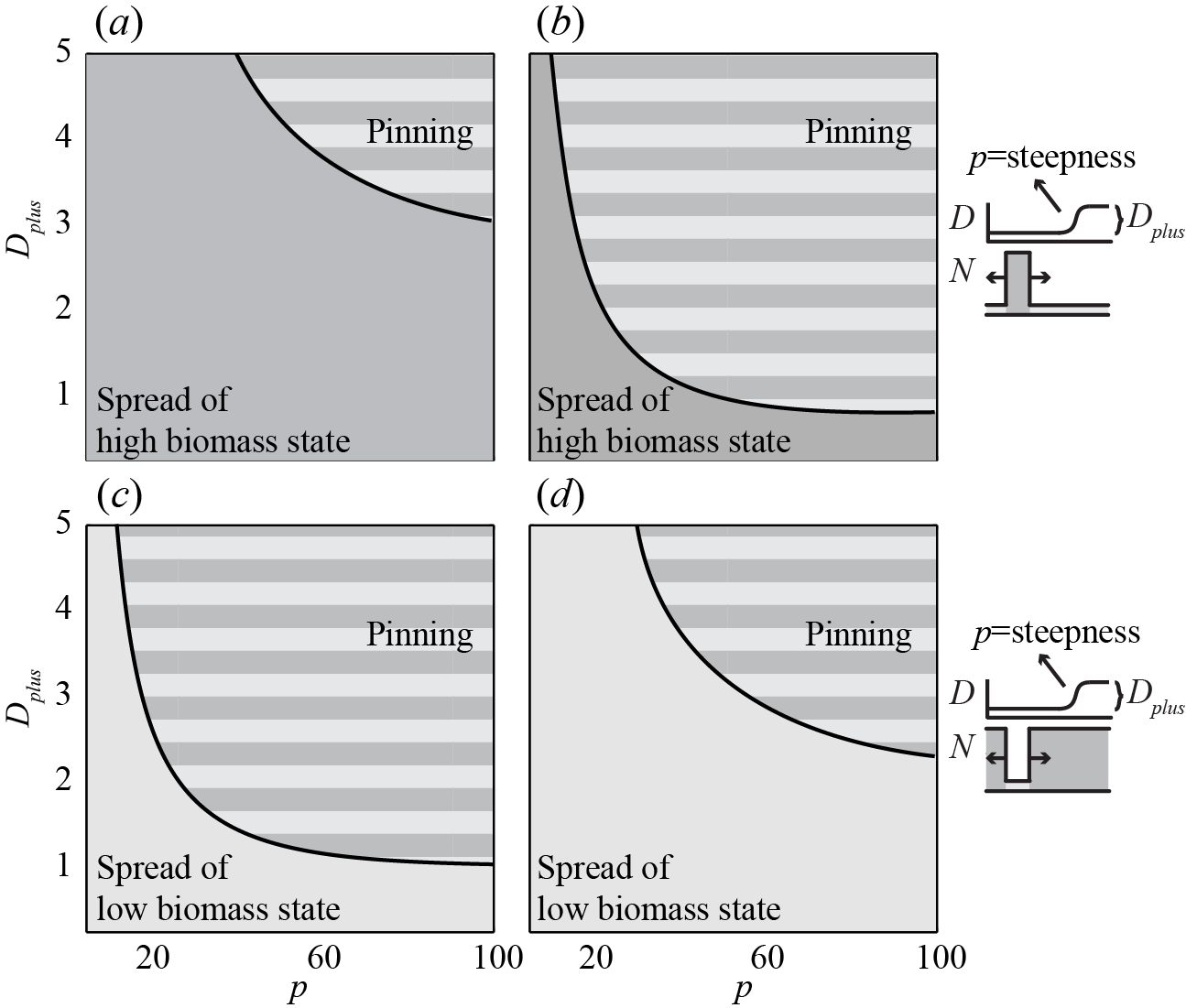

Supplement: S6 Fig — A travelling wave of collapsing biomass triggered by a disturbance can come to a halt if it meets an area of increased diffusion (D plus). The probability of this so-called pinning increases if the shift in diffusion becomes less gradual, thus if the steepness of the shift (p) increases. Maximal mortality rate: (a) c = 2.2; (b) c = 2.3; (c) c = 2.4 (d) c = 2.45. As shown by Fig. 5 (main text), the likelihood of pinning is high if the maximal mortality rate is close to the Maxwell point (e.g. panels (b) and (c)). (TIF) [file pone.0116859.s006.tif]

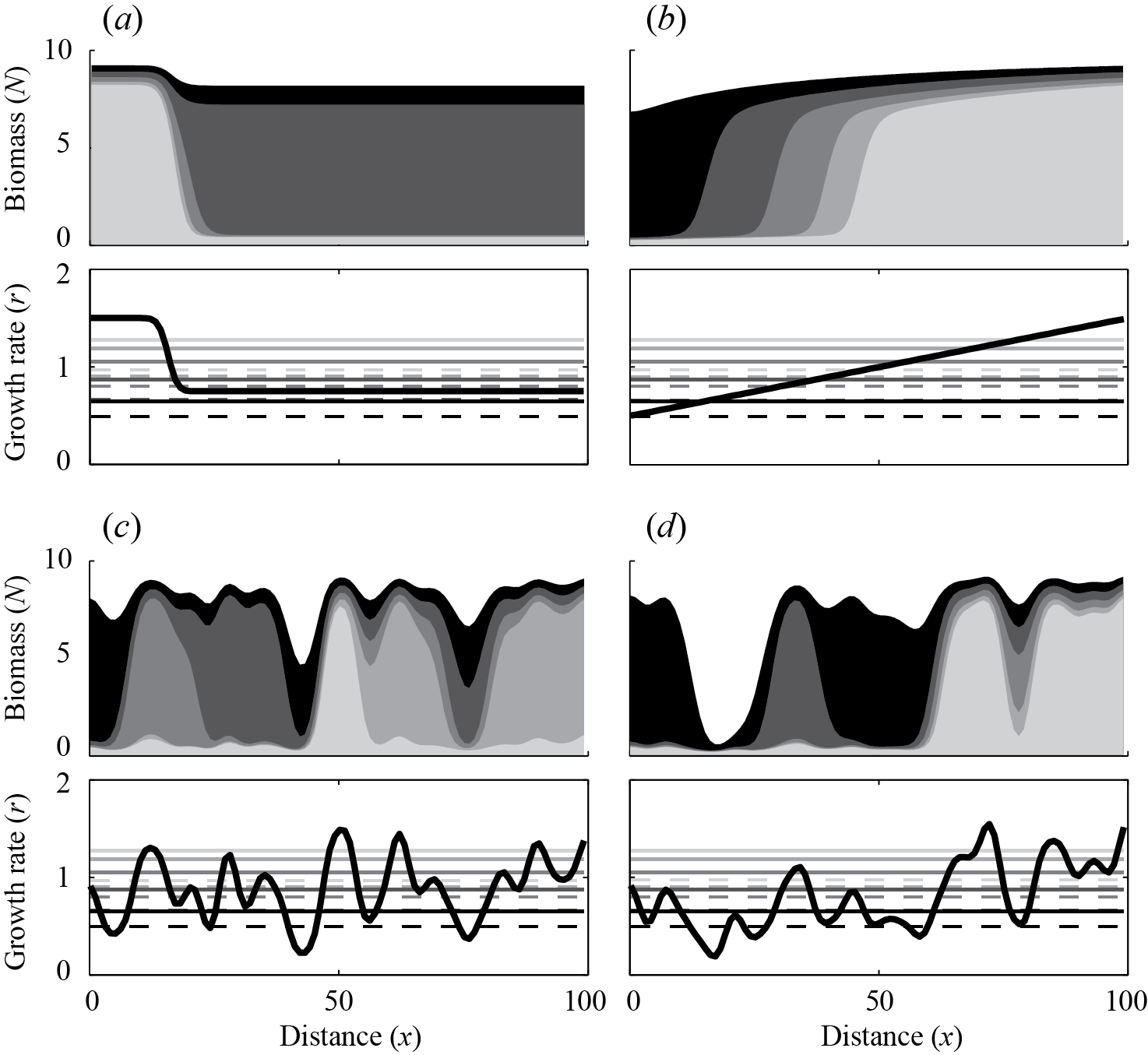

Supplement: S7 Fig — Example simulations of recovery, following a gradual decrease in maximal mortality rate on a landscape with local alternative stable states in: (a) a generally homogeneous landscape with one edge at which the growth rate is locally high (a refuge); (b) a landscape with a linear gradient in growth rate; and (c) and (d) two landscapes with random heterogeneity in growth rate. The thick black line in the lower panels represents the local growth rate r. The maximal mortality rate is changed from 2.36 to 1.96, and the simulation results are depicted for steps of 0.02. The stable end configurations of biomass are depicted in the upper panels as shaded areas, ranging from light gray to black. For each parameter setting, the grey solid lines in the lower panels represent the upper fold bifurcation, and the grey dashed lines the Maxwell point. When the growth rate at a point in space exceeds the fold bifurcation, there is locally only one stable state. When the growth rate locally exceeds the Maxwell point, a travelling wave towards the higher biomass could be triggered, provided that the area that initiates the wave is sufficiently large. Note that in an environment with an environmental gradient (panel b), the location of a standing wave directly follows changes in global conditions (no hysteresis), while in an environment with random heterogeneity, the location of the standing wave changes stepwise, which can result in hysteresis if the maximal mortality rate decreases again. (TIF) [file pone.0116859.s007.tif]
