# Supplementary material for: Resilience of Alternative States in Spatially Extended Ecosystems
Source: PLoS One. 2015 Feb 25;10(2):e0116859. doi: 10.1371/journal.pone.0116859 (PMC4340810; doi:10.1371/journal.pone.0116859)
Supplement: S1 Table — (DOCX) [file pone.0116859.s008.docx]

**Table S1.** Model equations and parameters of three models with alternative stable states.

| **Models** | **Definition and value** | | |  |
| --- | --- | --- | --- | --- |
| *Eutrophication model* [1]  $\frac{dN}{dt}=a-bN+\frac{rN^{p}}{N^{p}+1}+D\frac{\partial^{2}N}{\partial x^{2}}$ | *N*  *a*  *b*  *p*  *r*  *D* | Local nutrient concentration (state variable)  Nutrient loading rate  Nutrient loss rate  Hill coefficient  Maximum recycling rate  Nutrient mixing rate | 0.1-0.6  0.8  8  1  10 |  |
| *Allee effect model* [2] *with harvesting*  $\frac{dN}{dt}=rN\left( 1-\frac{N}{K} \right)\left( \frac{N}{K}-\frac{C}{K} \right)-fN+D\frac{\partial^{2}N}{\partial x^{2}}$ | *N*  *C*  *f*  *K*  *r*  *D* | Local population size (state variable)  Allee threshold  Exploitation rate  Local carrying capacity  Maximal growth rate  Dispersal rate | 0.2  0-0.2  1  1  10 | |
| *Vegetation-turbidity model* [3]  $\frac{dE}{dt}=E\left( 1-\frac{E}{E_{0}}\frac{h_{V}+V}{h_{V}} \right)+D_{E}\frac{\partial^{2}E}{\partial x^{2}}$  $\frac{dV}{dt}=\frac{r_{V}}{r_{E}}V\left( 1-V\frac{{h_{E}}^{p}+E^{p}}{{h_{E}}^{p}} \right)+D_{V}\frac{\partial^{2}V}{\partial x^{2}}$ | *V*  *E*  *E_0_*  *r_v_*  *r_E_*  *h_E_*  *h_V_*  *p*  *D_V_*  *D_E_* | Local vegetation cover (state variable)  Vertical light attenuation (state variable)  Background light attenuation  Maximum vegetation growth rate  Maximum turbidity  Half saturation turbidity level of the effect of extinction on vegetation growth  Half saturation vegetation cover of the effect of vegetation on light attenuation level  Hill coefficient  Dispersal rate vegetation  Mixing rate turbidity | 4-8  0.05  0.1  2  0.2  4  0.1  1 | |

1. Carpenter SR, Ludwig D, Brock WA (1999) Management of eutrophication for lakes subject to potentially irreversible change. Ecol Appl 9: 751–771.

2. Keitt TH, Lewis MA, Holt RD (2001) Allee effects, invasion pinning, and species’ borders. Am Nat 157: 203–216.

3. Scheffer M (1998) Ecology of shallow lakes. 1st ed. London: Chapman and Hall. 357 p.
