# Supplementary material for: Resilience of Alternative States in Spatially Extended Ecosystems
Source: PLoS One. 2015 Feb 25;10(2):e0116859. doi: 10.1371/journal.pone.0116859 (PMC4340810; doi:10.1371/journal.pone.0116859)
Supplement: S1 Text — (DOCX) [file pone.0116859.s009.docx]

**Text S1. Non-dimensional model**

We reduced the number of parameters by rescaling the model described in the main text:

$\frac{\partial N^{*}}{\partial t^{*}}=N^{*}(1-N^{*})-\frac{\sigma(N^{*})^{2}}{(N^{*})^{2}+\mu^{2}}+\frac{\partial^{2}N^{*}}{\partial(x^{*})^{2}}$
With new, dimensionless variables: $t^{*}=rt$, $N^{*}=\frac{N}{K}$, $x^{*}=x\sqrt{\frac{r}{D}}$, $L^{*}=L\sqrt{\frac{r}{D}}$
and parameters: $\sigma=\frac{c}{rK}$, $\mu=\frac{H}{K}$.

In this rescaled, non-dimensional model, the population size is scaled to the carrying capacity, the timescale is scaled to the growth rate of the species, and the spatial scale is scaled to the diffusion rate. Using such formulation, one can predict generic patterns independent of population size, temporal scale, and spatial scale. However, it is often more difficult to understand the abstract variables and parameters.

The non-dimensional model allows us, for example, to generalize the minimum size of a disturbance needed to trigger a travelling wave (as in Figure 3*a*, main text), in terms of the rescaled, non-dimensional perturbation size$\Delta x^{*}$ (Figure S4*a*). It also allows us to draw the hysteresis plot in terms of the rescaled landscape size *L* ­*(Figure S4*b*, as in Figure 4*b* and 4*c*, main text).
