# Supplementary material for: Resilience of Alternative States in Spatially Extended Ecosystems
Source: PLoS One. 2015 Feb 25;10(2):e0116859. doi: 10.1371/journal.pone.0116859 (PMC4340810; doi:10.1371/journal.pone.0116859)
Supplement: S2 Text — (DOCX) [file pone.0116859.s010.docx]

**Text S2. The Maxwell point in a range of bistable models**

Mathematically, one can describe the set of conditions at which the Maxwell point is found as follows [1–3](REFS: [Aronson and Weinberger, 1975](#_ENREF_1); [Fife, 1979](#_ENREF_8)):

with $f_{N}(N)$as the reaction part of the reaction-diffusion equation (e.g. equation 2, main text), and $\bar{N}_{1}$and $\bar{N}_{2}$ as the two alternative equilibria. To illustrate this, the reaction part (i.e. the growth function) of the exploitation model described in the main text is depicted in Figure S1. At the Maxwell point, the shaded areas are of equal size, so both states in the spatially extended system are equally resilient. If the mortality rate is lower, the high biomass state is more resilient, and a wave may be triggered to this state. Similarly, if the mortality rate is higher, the low biomass state is more resilient, and a wave may be triggered to this state.

The condition for the Maxwell point is generic for models with local alternative stable states and diffusion (Figure S2). Related to the existence of a Maxwell point is the effect that resilience drops at conditions equal to the Maxwell point if the size of the landscape is large (Figure S2). We used three different ecosystem models to illustrate this generality (Table S1): 1) a model with one state variable describing the eutrophication level in lakes. Nutrient dynamics are assumed to depend on nutrient input, loss (e.g. sedimentation, outflow), and recycling (e.g. from sediment or consumers) following a sigmoid function of the nutrient level [4] (Figures S2*a* and *b*), 2) a model with one state variable describing a population with an Allee effect [5], that is being harvested with a type I functional response (Figures S2*c* and *d*), and 3) a well-developed model with two state variables describing the dynamics of aquatic vegetation and vertical light attenuation in shallow lakes. Aquatic vegetation is assumed to have a positive feedback on its own growth, by reducing turbidity, which enhances vegetation growth [6] (Figures S2*e* and *f*).

**­** Interestingly, in contrast to models with one state variable, for models with two state variables the conditions for the Maxwell point change with diffusion rate. More specifically, in the vegetation-light attenuation model, the conditions for the Maxwell point depend on the dispersal rate of vegetation relative to the mixing rate of turbid water (Figure S3). Under the assumption that clonal expansion rate of aquatic plants is much lower than mixing of turbidity (i.e. *D_V_*/*D_E_* < 1), one may conclude that the resilience of the macrophyte-dominated state against local removal of vegetation in a large lake is not only lower than predicted by well-mixed models (see main text), but it is also lower than predicted by models that assume equal diffusion rates. Therefore, an increase in clonal expansion rate would lead to an increase in resilience of the macrophyte-dominated state.

**References**

1. Pomeau Y (1986) Front motion, metastability and subcritical bifurcations in hydrodynamics. Phys D Nonlinear Phenom 23: 3–11.

2. Fife P (1979) Long time behavior of solutions of bistable nonlinear diffusion equations. Arch Ration Mech Anal 70: 31–36.

3. Aronson DG, Weinberger HF (1975) Nonlinear diffusion in population genetics, combustion, and nerve pulse propagation. Lect Notes Math 446: 5–49.

4. Carpenter SR, Ludwig D, Brock WA (1999) Management of eutrophication for lakes subject to potentially irreversible change. Ecol Appl 9: 751–771.

5. Keitt TH, Lewis MA, Holt RD (2001) Allee effects, invasion pinning, and species’ borders. Am Nat 157: 203–216.

6. Scheffer M (1998) Ecology of shallow lakes. 1st ed. London: Chapman and Hall. 357 p.
