# Supplementary material for: Resilience of Alternative States in Spatially Extended Ecosystems
Source: PLoS One. 2015 Feb 25;10(2):e0116859. doi: 10.1371/journal.pone.0116859 (PMC4340810; doi:10.1371/journal.pone.0116859)
Supplement: S3 Text — (PDF) [file pone.0116859.s011.pdf]

Text S3. Co-existence of alternative stable states

Alternative stable states can co-exist in space, if the landscape consists of patches (Figure S5), or if there is some level of heterogeneity in the dispersal rate or mixing rate of the state variable (Figure S6).

Landscapes are often modeled by means of a lattice differential equation (LDE) [1]. For an LDE, one models the landscape as a grid with discrete grid cells. Dispersal is represented by an exchange of biomass between neighboring grid cells. This method is often used to mimic a continuous landscape, using sufficiently small grid cells relative to the dispersal rate, to avoid potential side effects of the ‘artificial’ grid cells. LDEs may also be used to simulate a patchy system, in which the grid cells represent individual patches, or islands. We made a simple LDE version of our model with ten grid cells, using:

$$\frac{dN_j}{dt} = rN_j\left(1-\frac{N_j}{K}\right)-\frac{cN_j^{\,2}}{N_j^{\,2}+H^2}+\frac{D}{\left(\Delta x\right)^2}\left(N_{j-1}+N_{j+1}-2N_j\right) \qquad j = 1...10$$

Using an LDE, it is possible to have areas with low biomass coexisting with areas with high biomass. Between both states there is a stagnant gradient in the biomass (Figure S5*a*). Such coexistence is only possible for intermediate harvesting rates and low dispersal rates between the grid cells (Figure S5*a*). The range of co-existence decreases with high dispersal. In contrast, if we model continuous space by means of a PDE (as in the main text), co-existence of alternative stable states is not possible for any parameter setting (Figure S5*b*). A travelling wave with a constant rate of spread always emerges, leaving the entire system in the state with the highest resilience (main text).

The prediction that alternative stable states cannot co-exist in space holds thus only for the limit case where exchange rates are completely homogeneous in space. As soon as that assumption is relaxed, alternative stable states can coexist in continuous space models if the exchange is sufficiently small (Figure S6). Co-existence of alternative stable states in continuous space may happen when a travelling wave meets an area of increased diffusion, such that the processes that stabilize the alternative state locally have a higher impact. This effect is most pronounced close to the Maxwell point, if processes that trigger the travelling wave are weak. Obviously, the more spatially heterogeneous the exchange rates or other spatial processes are, the larger the scope for such ‘wave-pinning’ situations (Figure S6).

A corollary of this result is that spatial coexistence of alternative stable states in finite element models (Figure S5*a*) is not simply an artifact of the discretization of space [2–5], but a consequence of

heterogeneous local exchange rates. This is important, as discrete models are much easier to implement computationally.

References

1. Chow S-N, Mallet-Paret J, van Vleck ES (1996) Dynamics of lattice differential equations. Int J Bifurc Chaos 6: 1605–1621.

2. Fáth G (1998) Propagation failure of traveling waves in a discrete bistable medium. Phys D Nonlinear Phenom 116: 176–190.

3. Keitt TH, Lewis MA, Holt RD (2001) Allee effects, invasion pinning, and species' borders. Am Nat 157: 203–216.

4. Holt RD, Keitt TH, Lewis MA, Maurer BA, Taper ML (2005) Theoretical models of species' borders: single species approaches. Oikos 108: 18–27.

5. Taylor CM, Hastings A (2005) Allee effects in biological invasions. Ecol Lett 8: 895–908.
