# Supplementary material for: Resilience of Alternative States in Spatially Extended Ecosystems
Source: PLoS One. 2015 Feb 25;10(2):e0116859. doi: 10.1371/journal.pone.0116859 (PMC4340810; doi:10.1371/journal.pone.0116859)
Supplement: S4 Text — (DOCX) [file pone.0116859.s012.docx]

**Text S4. Gradual shifts in heterogeneous spatially extended systems**

Abrupt shifts of alternative stable states in space may be expected if environmental conditions (here represented by the maximal growth rate *r*) change gradually in space (Figure S7). In such situation, a travelling wave triggered by a local disturbance will come to a hold if it hits environmental conditions that correspond to the Maxwell point (Figure S7). One may wonder what happens to the location of the spatial shift if global conditions change. We simulated the effect of an increasing globally changing driver (here represented by the maximal mortality rate *c*) in various spatially extended landscapes (Figure S7). A smooth environmental gradient of the maximal growth rate will simply result in a smooth range shift (Figure S7*b*), while a system with healthy edges may recolonize from these refuges (Figure S7*a*) as soon as the Maxwell point is crossed. In general, heterogeneity in spatially extended systems with local alternative states will lead to an overall smooth response to gradually changing conditions, with some larger waves of collapse or repair, when crossing more vulnerable, or healthy parts of the landscape (Figures S7*c* and S7*d*). The Maxwell point plays an important role, as it is the point at which the direction of the travelling wave shifts: it determines the borders of spatial co-existence of alternative stables states.
